# Dynamic pricing modeling and inventory management in omnichannel retail using Quantum Decision Theory and reinforcement learning

**DOI:** 10.1371/journal.pone.0333068

**Published:** 2025-10-21

**Authors:** Shima Roosta, Seyed Jafar Sadjadi, Ahmad Makui

**Affiliations:** School of Industrial Engineering, Iran University of Science and Technology, Tehran, Iran; Symbiosis International (Deemed University), INDIA

## Abstract

In the world of omnichannel retail, where customers seamlessly switch between online and offline channels, pricing and inventory management decisions have become more complex than ever. Customer purchasing behavior is influenced by uncertainty, market fluctuations, and competitive interactions, which traditional models fail to accurately predict. In such conditions, the need for intelligent and adaptive decision-making frameworks is more critical than ever. For the first time, this study presents a novel approach combining Quantum Decision Theory, Quantum Markov Chains (QMC), Quantum Dynamic Games, and Reinforcement Learning to optimize dynamic pricing and inventory management. By leveraging concepts such as superposition, observer effect, and quantum interference, the proposed model overcomes the limitations of classical models and provides a deeper understanding of customer behavior in uncertain environments. Additionally, a Quantum Multi-Level Markov Process (QMLMP) is employed to model market variations and enhance predictions. The results of this study demonstrate that the innovative model improves the accuracy of purchase behavior predictions, optimizes pricing and inventory management strategies, and helps retailers make more competitive and profitable decisions. This research introduces a transformative approach to tackling retail challenges in the digital age and paves the way for future studies in this domain.

## 1. Introduction

The retail industry is considered one of the most important and active sectors of the global economy. In the United States, this industry contributes $3.9 trillion to the annual Gross Domestic Product (GDP) and supports one-quarter of American jobs (Rios and Vera, 2023). E-commerce has brought transformations in omnichannel retailing. Many traditional retailers are entering the digital world. By adopting dual-channel and multi-channel strategies, retailers seek to increase their market share. On the other hand, customers no longer differentiate between various channels and expect to seamlessly switch between online and offline platforms. As a result, the boundaries between physical and digital channels are fading, and retail is moving towards a seamless omnichannel experience [1]. For example, Walmart has not only launched online stores but has also partnered with e-commerce platforms. Meanwhile, many online retailers are expanding their physical presence. For instance, in October 2016, The Idle Man, a UK-based online menswear retailer, opened its first physical store in London [1]. From the customer’s perspective, the omnichannel model enables shopping through various methods, such as buying online and picking up in-store, buying online and shipping from a store, reserving online and purchasing in person, and in-store shopping with home delivery. However, from the retailer’s perspective, these changes present both opportunities and challenges, as they require a reevaluation of sales channel structures, strategies, and operational decisions [1]. Omnichannel retailers must make a wide range of decisions that impact their profitability and competitiveness.

Demand is one of the key factors in sales for any organization or company. Researchers have proposed dependent demand functions for modeling demand, such as the price-dependent demand function [2,3] and the inventory-dependent demand function [4,5]. Therefore, the joint management of pricing and inventory is considered a fundamental issue in supply chain management [6]. Numerous studies have simultaneously addressed the importance of these two factors, and interested readers can refer to them [7–12].

Pricing of products is one of the most important decisions for companies and organizations. Setting the right price for products and services significantly impacts profitability and business success. According to Utility Theory, customers seek to maximize utility in their purchasing decisions; therefore, pricing should align with their perceived value of the product. In other words, the price acceptance level represents the maximum amount a customer is willing to pay for a product or service, and price acceptance is one of the consequences of customer satisfaction [13,14]. Furthermore, the Price Elasticity of Demand theory indicates that customers respond differently to price changes. For some products, a price reduction leads to increased demand, while for others, price changes have a minimal impact. For this reason, multichannel retailers should adjust their pricing strategies based on the demand elasticity in each channel [15].

According to Reference Price Theory, customers have a reference price in mind for each product, which is shaped by past experiences and advertisements. If a product’s price exceeds the customer’s mental reference price, demand may decrease. Therefore, maintaining price consistency across different channels and using targeted discounts can be effective in attracting customers [16]. The impact of price on customers’ purchasing decisions is crucial, as appropriate pricing can either encourage customers to buy or deter them from making a purchase. Customers generally seek good value for their money—if the price of a product does not match its perceived value and quality, they may look for alternatives. Moreover, discounts and price increases can directly influence customer decisions. Proper pricing strategies help improve business profitability: if the price is too high, sales volume may decrease, reducing overall profit; conversely, if the price is too low, profit margins may shrink. Therefore, setting a balanced and strategic price is essential for sustained profitability.

Unlike traditional retail, where pricing is determined independently for each store, online customer interactions have blurred physical boundaries, making pricing strategies more complex. Multichannel retailers must consider inventory and demand across the entire network to determine optimal pricing for each channel and geographic region. Implementing unified pricing and joint order fulfillment policies can reduce the average order processing cost compared to separate pricing and fulfillment strategies [7]. E-commerce retailers employ various marketing strategies to boost sales. One common strategy is offering price discounts by temporarily lowering the regular price of a product [14]. Additionally, most retailers adopt dynamic pricing policies, where the initial price changes based on sales performance over time. The main pricing challenges in retail include: Monitoring and analyzing sales behavior over time and adjusting prices on a daily, weekly, or monthly basis. Coordinating prices across different store locations to prevent arbitrage, where resellers exploit price differences between stores. Managing inventory while considering product substitutes and complements. Optimizing final prices for remaining stock at the end of a sales period [17].

Inventory management in omnichannel retailing is a constant challenge. The efficiency and profitability of retail businesses largely depend on proper inventory management. Inventory-related issues play a crucial role in supply chain and logistics, as they directly impact a company’s financial performance. This area involves various challenges, such as reducing lost sales due to stock shortages, managing perishable products, and controlling inventory with random replenishment times [17,18]. Inventory levels also influence customer purchasing behavior—particularly when stock is low, customers may feel a greater sense of urgency to buy. One of the key challenges in pricing and inventory management in omnichannel retailing is complex customer behavior, market fluctuations, and uncertainty in purchase decisions. Customers expect consistent pricing across all channels, yet intense competition and rapid market changes make dynamic pricing essential.

Additionally, accurate demand forecasting is difficult due to fast-changing trends, seasonal shopping patterns, and diverse product options. As a result, inventory management becomes increasingly complex. To mitigate rising costs, prevent customer dissatisfaction, and maintain profitability, retailers must leverage data analytics and advanced technologies to optimize their pricing and inventory strategies.

Selecting the optimal pricing strategy and inventory management approach requires accurate forecasting of customer behavior. However, real customer behavior is often irrational and unpredictable. Traditional models, such as Markov models and classical reinforcement learning, assume that customers always make rational decisions. However, recent research has shown that this assumption is not always valid.

Recent research in cognitive psychology focuses on probabilistic theories that best describe human decision-making uder uncertainty. Three main theories have been proposed in this field: Classical Probability Theory (CPT): Assumes rational and logical decision-making. Heuristics and Biases: Describes human behavior based on intuitive and fast decision-making rules. Quantum Probability Theory (QPT): A novel approach for analyzing uncertain decision-making, which can address the limitations of CPT in explaining customer cognition. Traditional pricing and inventory models, such as those based on classical Markov processes or reinforcement learning, often assume that customers make decisions rationally, consistently, and based on utility maximization principles. However, empirical observations in omnichannel retail environments reveal that real-world customers often behave unpredictably. For example, a customer may view a product multiple times, abandon it, and then suddenly decide to purchase after seeing a social media post or a temporary discount. These irregularities — driven by emotions, external stimuli, and mental hesitation — cannot be adequately modeled using classical deterministic frameworks. Such models treat indecisive behavior as disinterest and ignore the dynamic, context-sensitive nature of customer cognition. Therefore, a more flexible and realistic approach is needed. Quantum Decision Theory (QDT) provides this by allowing for probabilistic states of decision-making, where a customer can simultaneously exist in both “buy” and “not buy” cognitive states until an external interaction collapses this uncertainty into a final decision. This quantum perspective better captures the ambiguity, non-linearity, and interference effects inherent in modern consumer behavior.

Quantum Probability Theory (QPT) has emerged as a new paradigm in recent years, gaining widespread application in psychology, cognitive science, and decision-making analysis [19]. Cognitive quantum research suggests that QPT can better explain cognitive biases and decision-making deviations in customers. While CPT assumes that a customer always exists in one specific state, QPT allows a customer to exist in multiple cognitive states simultaneously. QPT provides a mathematical probabilistic model where a customer can be in an infinite number of potential states at any given moment, accessing all possible options [20]. In recent years, quasi-quantum decision theories have demonstrated their ability to explain inconsistencies in human behavior using quantum probabilities [21]. Quantum Decision Theory (QDT) introduces a new framework for modeling customer behavior, naturally incorporating cognitive uncertainty. In this framework, customers remain in multiple probabilistic states until they make a final decision. Their final choice is influenced by quantum superposition and interference effects. Unlike classical models, which assume that a customer either purchases or does not purchase, the quantum model allows a customer to be in both states (buying and not buying) simultaneously—until an external interaction (such as a price change or advertisement) triggers their final decision.

Customer Lifetime Value (CLV) is an important concept in marketing management and customer relationship management, representing the profit a customer generates for a business over their lifetime. This metric is crucial for organizations because retaining existing customers is less costly than acquiring new ones. Considering CLV is particularly important when delivering goods to various regions. If only current demand is considered, businesses may meet short-term needs but miss out on long-term growth opportunities. By focusing on CLV, companies can look to the future and make decisions that help retain customers and increase profitability in the long run. Customers with high CLV tend to make more purchases and generate higher profits for the company. Therefore, even if current demand is low in some regions, paying attention to CLV can help businesses optimally allocate resources and provide better services to these customers, keeping them loyal [22–24].

Customer satisfaction and loyalty are key factors that lead to business success in competitive markets [25,26]. Customer loyalty refers to something that influences customer behavior to ensure ongoing, long-term purchases. The costs of acquiring new customers are higher than retaining them, so service organizations must find ways to retain their customers. Loyal customers buy from a business and may recommend it to others, but this does not mean they are always satisfied. For example, disloyal customers may change their purchasing location if a better option is available. However, satisfied customers may continue to buy from their current suppliers. The consumer is the core of organizations around which marketing activities revolve. Retaining consumer loyalty is what organizations aspire to achieve. The highly competitive and broad market with numerous brands is a harsh reality that marketing managers face today, and acquiring a new customer is more expensive than serving an existing one. Therefore, consumer loyalty is considered the most desirable phenomenon for companies, as commitment leads to repeat buying behavior and positive word-of-mouth advertising, thus resulting in a more significant market share and superior financial performance for the company [27].

Dynamic games are a powerful tool for modeling interactions between economic agents over time. This framework is particularly useful in scenarios where past decisions influence the current state and future choices. In the field of retail and dynamic pricing, numerous studies have utilized dynamic game theory to analyze optimal pricing strategies, inventory management, and customer responses [8,28,29].

The Markov Decision Process (MDP) is a mathematical model used for decision-making in stochastic or uncertain environments. This model is specifically designed for problems where the decision-maker cannot predict future outcomes with certainty. MDP has significant applications in fields such as reinforcement learning, robotics, game design, and resource management. Many studies have applied MDP-based approaches to dynamic pricing and inventory control [30,31]. In general, MDP provides a mathematical framework for decision-making in uncertain environments, where state transitions occur probabilistically based on the actions taken. This model is particularly useful for analyzing dynamic games, where state changes occur in a probabilistic manner.

Common dynamic pricing methods include Markov chains, reinforcement learning (PPO, Q-learning), and dynamic game theory. These methods have been successful in various economic problems but have limitations. They assume that customers always make rational and deterministic decisions, whereas in reality, purchase decisions are influenced by emotions, social factors, and random interactions. This scientific gap creates the need for a new approach that can account for these complexities in modeling. Traditional pricing and inventory management models, based on Markov chains and classical reinforcement learning, assume that customers always make logical and definite decisions. However, recent research in quantum cognition suggests that customer decision-making can exist in multiple cognitive states simultaneously and be influenced by quantum superposition. Despite this breakthrough, few studies have applied this concept to omnichannel retailing and dynamic pricing. Recent findings in psychology and behavioral economics show that customer decisions are affected by irrational factors, emotions, advertisements, and even discounts. These unexpected and fluctuating behaviors cannot be accurately modeled using classical methods. Thus, there is a growing need for a new framework to analyze and predict customer behavior in omnichannel retailing.

This scientific gap led us to explore Quantum Markov Chains (QMC) and Reinforcement Learning (RL) to better model customer purchasing behavior and optimize pricing strategies and inventory management. In this study, we use Quantum Decision Theory (QDT) to model customer purchasing behavior. Unlike classical models, quantum theory enables more accurate predictions of customer behavior. In this framework, decisions are modeled using quantum concepts such as superposition and the observer effect, rather than traditional probabilities. In this model, customers can exist in multiple decision states simultaneously until an external factor, such as an advertisement or price change, collapses the state and determines their final decision. This research aims to answer: How can Quantum Decision Theory, combined with Reinforcement Learning, optimize pricing and inventory management in omnichannel retailing? Specifically, we focus on:

1. Quantum Markov Chains (QMC) for predicting customer purchasing behavior.
2. Quantum probabilities in pricing models, integrating price dynamics with market conditions.
3. Reinforcement Learning (RL) to optimize pricing and inventory management policies.
4. Pricing formulation using Schrödinger-like quantum equations.
5. Modeling customer-seller interactions as a Quantum Game Theory framework.
6. Using Quantum Markov Chains (QMC) to enhance purchasing behavior predictions.

This study introduces a new framework for dynamic pricing and inventory management in omnichannel retail by integrating quantum-based models with machine learning techniques. Our proposed model, leveraging quantum concepts, overcomes the limitations of traditional methods such as classical Markov chains and classical reinforcement learning. This approach better models the cognitive uncertainty of customers, leading to more accurate predictions of purchasing behavior. Furthermore, by integrating quantum probabilities and reinforcement learning (RL), our model enables the simultaneous optimization of pricing and inventory management, ultimately enhancing the profitability of retailers in an omnichannel environment. The remaining structure of the paper is as follows: In Section 2, the literature review is presented. In Section 3, the mathematical modeling is introduced. In Section 4, the solution method is examined. In Section 5, the implementation and execution process of the model is discussed. In Section 6, the results of the model are presented. In Section 7, a comparison of the model with traditional models is made. Section 8 covers sensitivity analysis, and Section 9 provides the conclusion and outlines future research directions.

## 2. Literature review

Joint pricing and inventory management is a fundamental issue in supply chain management [6]. With the expansion of supply chains and the rise of e-commerce and omnichannel retailing, this area has garnered increasing attention, leading to numerous studies. Managing pricing and inventory in omnichannel retailing presents significant challenges due to the complex interactions between sales channels, the uncertain behavior of customers, and intense competition. Many studies have attempted to model these challenges using classical approaches, such as Markov chains, reinforcement learning (RL), and game theory. To address the complexities of pricing and inventory management, researchers have increasingly relied on reinforcement learning (RL) and advanced algorithms like Proximal Policy Optimization (PPO) and Q-learning [11,30]. These models can learn optimal policies in dynamic environments, outperforming traditional models. However, reinforcement learning models still assume rational customer behavior. Recent research has shown that customers exhibit unpredictable behaviors under uncertainty, which cannot be fully captured by traditional RL models.

Dynamic pricing has emerged as a key strategy for increasing profitability in retail. Many studies have utilized Markov chains and the Markov Decision Process (MDP) to determine optimal pricing policies [7,8]. These models assume that the decision-maker can observe the market conditions and, based on this information, adopt optimal pricing strategies. However, these models have a fundamental limitation: they assume that customers always make rational decisions based on economic utility, whereas behavioral psychology studies have shown that purchase decisions are influenced by irrational factors, such as emotions, mental perceptions, and social effects [32,33].

Cognitive psychology research has shown that customer decision-making is not always rational and is often influenced by factors such as intuition, emotions, and social effects [34,35]. One of the emerging theories proposed to explain these behaviors is Quantum Probability Theory (QPT), which, instead of deterministic models, uses superposition and probabilistic interference to predict decisions [20]. While QPT has been successful in cognitive sciences and psychology, few studies have applied this approach to retail and dynamic pricing. This paper aims to bridge the gap in customer behavior modeling by utilizing quantum modeling, dynamic games with quantum concepts, and Quantum Markov Chains (QMC). Table 1 presents a summary of research on dynamic pricing and inventory management.

**Table 1:** Literature Review.

Research Paper	Pricing	Inventory	Channel	Certainty or uncertainty		QPT	Problem Type	Method
				in customer behavior	Other			
[36]	✓	✓	Single	×			Static	
[37]	✓	✓	Single	×	×		Static	Mathematical Modeling
[38]	✓	✓	Single	×	✓		Dynamic	Stackelberg game theory model + numerical algorithm
[5]	✓	✓	Single	×	×		Dynamic	Theoretical analysis + numerical example
[39]	✓	✓	Single	×	✓		Dynamic	Data-Driven Nonparametric Algorithm
[39]	✓	✓	Single	×				
[21]	✓	✓	Single	×				DRL
[31]		✓	Omni	×			Dynamic	
[28]	✓	✓	Single	×			Dynamic	
[19]		✓	Single	×				DRL
[30]	✓	✓	Single	×				DRL
[40]	✓	✓	Single	×	×		Static	Analytical Method
[17]	✓	✓	Multi	×	✓		Dynamic	Robust Stochastic Optimization
[6]	✓	✓	Omni	×				DRL
[1]	✓	✓	Omni	✓	✓		Dynamic	Augmented ε-constraint method
[41]	✓	✓	Omni	×	✓		Dynamic	Robust Optimization
[42]	✓	✓	Multi	×	✓		Dynamic	Robust Optimization
[43]	✓	✓	Dual	×	✓		Dynamic	Game Theory
[44]	✓	✓	Multi	✓	✓		Static	Optimization models or economic analyses
Current Research	✓	✓	Omni	✓	✓	✓	Dynamic	PPO

By reviewing the literature, it became evident that traditional models, such as Markov processes and reinforcement learning in dynamic pricing and inventory management, assume that customers act rationally. However, recent research in cognitive sciences has shown that customer decisions can be irrational and uncertain. In this paper, for the first time, quantum modeling, quantum dynamic games, and Quantum Markov Chains (QMC) are utilized to model customer purchasing behavior and optimize pricing and inventory policies in omnichannel retail. This approach can enhance prediction accuracy, improve decision-making efficiency, and provide innovative solutions for sales management under uncertainty.

## 3. Modeling the omnichannel retail problem using quantum decision theory

This study focuses on modeling an omnichannel retail system that integrates both physical stores and online sales. The primary goal of this modeling approach is to optimize inventory management and pricing strategies in a way that not only maximizes retailer profit but also increases the likelihood of customer purchases. To achieve this objective, the proposed model follows a bi-objective approach, where a dynamic mathematical model is designed to simultaneously analyze multiple parameters, including costs, demand, customer lifetime value (CLV), and purchasing behavior. This model particularly emphasizes both online and physical demand and considers two types of demand [1]:

1. Physical Demand – Demand generated at physical stores, each corresponding to a specific market region.
2. Online Demand – Customers can make purchases through the retailer’s website and choose from various delivery options, such as standard delivery, in-store pickup, or instant delivery.

With the expansion of omnichannel retail, pricing and inventory management have become challenging due to the complex and uncertain behavior of customers. Traditional models, such as Markov chains and classical reinforcement learning, assume that customers always make rational and deterministic decisions. However, recent studies have shown that purchase decisions are influenced by uncertainty, emotions, and environmental factors. Quantum Decision Theory (QDT) is a novel mathematical framework that can model human decisions in a non-classical manner. Unlike classical models, which assume that decisions are made deterministically and rationally, QDT demonstrates that human decisions, especially under uncertainty, follow quantum principles. Additionally, in the study by [45], it is argued that mathematical structures derived from quantum theory provide a much better explanation of human thinking than traditional models. Moreover, in the research by [46], the preference reversal phenomenon in Quantum Decision Theory (QDT) is examined, showing how quantum interference effects can explain cognitive biases, particularly in cases where classical decision-making theories fail. To address this issue, we utilize Quantum Decision Theory (QDT), which incorporates concepts such as superposition, the observer effect, and Quantum Markov Chains (QMC) to more accurately model customer behavior. In the proposed model, customer purchase behavior is represented as a probabilistic state rather than a fixed decision. According to the principle of superposition, a customer remains in multiple possible states (buying or not buying) until an external factor—such as an advertisement or price change—collapses the state into a definite decision (observer effect). Additionally, the interference effect shows that customer choices influence each other in unexpected ways.

**Superposition:** In quantum mechanics, a system can exist in multiple possible states simultaneously until an observation or measurement forces it into one definite state. In the customer purchase model, this concept suggests that a customer may simultaneously consider both buying and not buying a product. Only after seeing an advertisement or a price change does the customer finalize their decision. To better grasp our proposed model and how we apply **quantum concepts** to analyze customer behavior, let’s simplify it. Take **superposition** as an example: we assume that until a customer makes their final decision, they exist in a state of both **buying and not buying** simultaneously. This means the customer is in both mental states at once, and their final decision only becomes clear after they’re exposed to an external factor, like seeing a new price or an advertisement.

**Observer Effect:** This principle states that observing or interacting with a system can alter its state. In retail, seeing advertisements, customer reviews, or special offers can shift the customer’s mental state, influencing them toward making a purchase decision [47]. To better understand, we can explain the **Observer Effect** in quantum theory like this: **observation or interaction with a system changes its state.** For instance, every time a customer sees a new advertisement, user review, or price change, their mental state shifts, and the probability of them buying or not buying undergoes a transformation.

**Interference Effect:** In quantum physics, quantum waves can interfere with each other, altering the final outcome. In the proposed model, if a customer is undecided between multiple options, advertisements and interactions can unexpectedly shift their final decision. For example, if a customer is considering two similar products, a discount or an advertisement on one product can increase its likelihood of being chosen [48]. To better understand, we can say that in **Interference**, a customer’s different choices (like choosing between two brands or two products) can quantum mechanically interfere with each other. This means if a customer is thinking about two options simultaneously, these two probabilities can either strengthen or weaken each other. For example, an advertisement for one product might cause the probability of buying a rival product to decrease.

**Wave Function:** In quantum mechanics, the wave function represents all possible states of a system. In the proposed model, the customer’s wave function reflects the probabilities of different purchasing decisions. When the customer sees an ad or experiences a price change, their wave function collapses, resulting in a definite purchasing decision [49].

Considering the discussions presented in the introduction, not all customers hold the same level of importance, and they do not generate equal profit for the organization. Therefore, taking into account Customer Lifetime Value (CLV) and customer loyalty is crucial when determining pricing and inventory for retail stores, which will be considered in this study. Accordingly, the pricing and inventory model incorporating quantum characteristics, along with CLV and customer loyalty, will be structured as follows:

**Table:** 

Quantum Variables
ψ_b_(t): Quantum Wave Function of Customer Behavior: The wave function representing customer behavior, influenced by price, inventory, demand, and emotions. A wave function representing customer behavior based on price, inventory, demand, and emotions. This function describes the probability of a customer being in different purchasing states and represents the customer’s mental state.
Possible customer states (based on purchase or non-purchase): ⟨purchase| ⟨non−purchase| ⟨delay in purchase|
P(i,j,k,r): The classical probability of the state (i, j, k, r) The probability of a customer being in a particular purchasing state, such as choosing brand **i**, product category **j**, geographical region **k**, and purchasing method **r**.
$\psi_{\text{b}}(\text{t}|\text{i},\text{j},\text{k},\text{r}\;)$: A wave function that represents the impact of various factors (price, advertising, inventory, and environment) on customer decision-making.
$\hat{H}$: A factor controlling system changes, which in this context includes the effects of price, advertising, inventory, and environmental factors.
${\hat{\text{H}}}_{\text{p}}$: The effect of price changes on customer behavior
${\hat{\text{H}}}_{\text{q}}$: The effect of inventory level on customer behavior
${\hat{\text{H}}}_{\text{d}}$: The effect of market demand on purchasing decision
${\hat{\text{H}}}_{ext}=\lambda \text{A}$: The impact of advertising and external factors (where λ is the advertising intensity coefficient and λA is the advertising intensity at time t)
$\text{i}\hbar \frac{\text{d}}{\text{dt}}\psi_{\text{b}}(\text{t}):\;\;$ Rate of Change of the Customer’s Wave Function Over Time:

**General Wave Function:** The general wave function is defined as a combination of classical and quantum variables. This wave function dynamically evolves based on customer purchasing decisions:


$$\psi_{\text{b}}(\text{t})=\sum_{\text{i},\text{j},\text{k},\text{r}}\psi_{\text{b}}\left(\text{t}|\text{p},\text{q},\text{d}\;\right).\text{P}(\text{i},\text{j},\text{k},\text{r}).\psi_{\text{b}}(\text{t}|\text{i},\text{j},\text{k},\text{r}\;)$$

**Wave Function Normalization Condition:**


$$\sum_{\text{b}}{\left|\psi_{\text{b}}(\text{t})\right|}^{\text{2}}=\text{1}$$

This equation is chosen to model customer decision-making in uncertain environments. The combination of the wave function and conditional probabilities allows for the simultaneous consideration of multiple influencing factors. This model dynamically represents the customer’s state transitions. If P(i,j,k,r) increases (for example, if a significant discount is applied to brand ii), the probability of selecting that brand increases. Viewing advertisements can alter the wave function and cause its collapse into a specific decision.

**Customer Behavior Change Equation:** To model customer behavior changes over time, we use the Schrödinger equation, where the wave function $\psi_{\text{b}}(\text{t})$ is influenced by price, inventory, demand, advertising, and external factors.


$${\hat{\text{H}}}_{\psi_{\text{b}}(\text{t})}=\text{i}\hbar \frac{\text{d}}{\text{dt}}\psi_{\text{b}}(\text{t})$$

$$\hat{\text{H}}={\hat{\text{H}}}_{\text{p}}+{\hat{\text{H}}}_{\text{q}}+{\hat{\text{H}}}_{\text{d}}+{\hat{\text{H}}}_{\text{ext}}$$

$${\hat{\text{H}}}_{\text{p}}=\;{\gamma \gamma }_{\text{1}}.{\text{p}}_{\text{ijkr}}\;(\text{t}),{\hat{\text{H}}}_{\text{q}}={\gamma \gamma }_{\text{2}}.{\text{S}}_{\text{k}}\;(\text{t}),{\hat{\text{H}}}_{\text{d}}=\;{\gamma \gamma }_{\text{3}}.{\text{d}}_{\text{ijk}}\;(\text{t}),{\hat{\text{H}}}_{\text{ext}}=\;\lambda \lambda \text{A}(\text{t})$$

This equation is one of the fundamental equations of quantum mechanics, used to model system changes over time. In this model, customer decisions evolve over time, and a final decision is made only when an observation (such as an advertisement or a price change) occurs. Price, inventory, advertising, and demand are among the most influential factors in customer purchases. Research has shown that these factors play a crucial role in customer decision-making [13] and [15]. This model is proposed based on the concepts of Quantum Decision Theory; however, it still requires empirical testing on real-world data. Some studies, such as [45] and [46], have explored modeling human cognitive behavior using quantum theory.

**Numerical Example for Better Understanding of the Model**: Suppose a store sells two types of products, A and B, in two regions, C and D. The advertising level and price of these products are as follows:

**Table:** 

Product	Price (USD)	Advertising Level	Inventory Level	Market Demand
A	100,000	Medium	50 units	High
B	120,000	High	30 units	Medium

Examining Changes in Customer Wave Function Over Time with Advertising

Before Advertising:


$$\varphi_{A}(\text{0})=\text{0}.\text{4},\varphi_{B}(\text{0})=\text{0}.\text{6}$$

The customer is more inclined to purchase Product B.

After Intensive Advertising for Product A:


$$\varphi_{A}(t)=\text{0}.\text{7},\varphi_{B}(t)=\text{0}.\text{3}$$

Increased advertising has changed the wave function, thereby altering the customer’s choice.

Price Change Analysis

If the price of Product A decreases:


$${\hat{\text{H}}}_{\text{p}}=\gamma_{\text{1}}\times (\text{100}\to \text{80})$$

This results in an increase in $\varphi_{A}(t)$, leading to a higher probability of purchasing Product A.

**Quantum and Classical Interactions:** To incorporate quantum and classical interactions, the purchase probability is modeled as follows:


$${\text{prob}}_{\text{ijk}}(\text{t})=\frac{\text{1}}{\text{exp}-\left(\alpha_{\text{1}}{\text{p}}_{\text{ijkr}}(\text{t})+\alpha_{\text{3}}.{\text{S}}_{\text{k}}(\text{t})+\alpha_{\text{4}}.{\text{d}}_{\text{ijk}}(\text{t})+\alpha_{\text{5}}.{\text{CLV}}_{\text{k}}(\text{t})+\sum_{\text{m}}\left(\text{quantum}\_\text{interaction}(\text{m})\right)\right)+\text{1}}$$

where quantum_interaction(m) represents the quantum effects on customer behavior. This function captures how quantum concepts such as **superposition**, **observer effect**, and **interference** influence customer decision-making.


$${\text{quantum}}_{\text{interaction}(\text{m})}=\left(P(m)-\text{1}\right).\beta_{\text{2}}.e^{-\gamma x_{m}}-\beta_{\text{1}}P(m)$$

where:

- P(m) is the probability of customer **m** making a purchase, calculated based on historical data or a logistic model.
- P(m)−1 represents the probability of the customer not purchasing.
- $x_{m}$ is an environmental variable that accounts for factors such as advertising impact, pricing, and special offers.
- $e^{-\gamma x_{m}}$ is an exponential function that moderates the effect of discounts or advertising, meaning that if the impact of advertising or pricing is high, this value decreases.
- $\beta_{\text{1}},\beta_{\text{2}}$ are coefficients that regulate classical and quantum effects.
- γγ controls the sensitivity of the customer to environmental variables.

**Mathematical Model:**

**Table:** 

Sets	
i∈I(t)	Product Set
j∈J(t)	Brand Set
k∈K(t)	Region Set
r(t)	Type of purchase or, in other words, method of sale (DC, Store, Pickup, Express)
**Parameters**	
Loyalty(k,t)	Dynamic customer loyalty in region k at time step t
β	Customer sentiment impact weight
δ	Customer loyalty impact weight
Θ	It is a coefficient that scales loyalty to time.
${\text{CLV}}_{\text{k}}(\text{t})$	Customer lifetime value in region k at time step t
${\text{S}}_{\text{k}}(\text{t})$	Predicted customer sentiment analysis in region k (from data) at time step t
${\text{c}}_{\text{ij}}(\text{t})$	Purchase cost of product i from brand j at time step t
${\text{cap}}_{\text{k}}(\text{t})$	Warehouse capacity in region k at time step t
B(t)	Total budget of the distribution center
${\text{d}}_{\text{ijk}}(\text{t})$	Total forecasted demand for product i from brand j in region k at time step t across all sales methods
${\text{dd}}_{\text{ijkr}}(\text{t})$	Forecasted demand for product i from brand j in region k using sales method r at time step t
$\alpha_{\text{1}},\alpha_{\text{2}},\ldots ,\alpha_{\text{7}}$	Weight coefficients for probability function
$\gamma_{\text{1}},\gamma_{\text{2}},\gamma_{\text{3}}$	Weight coefficients for dynamic pricing
$\kappa_{\text{1}},\kappa_{\text{2}},\kappa_{\text{3}}$	Weight coefficients for product shipment to different regions
${\text{initial}}_{\text{ijk}}$	Initial stock of product i from brand j in region k
$\alpha_{\text{6}}$	A weighting coefficient that determines the impact of the customer preference list on product selection
**Decision variables**	
${\text{prob}}_{\text{ijk}}(\text{t})$	Probability of purchasing product i from brand j in region k at time step t
${\text{p}}_{\text{ijkr}}(\text{t})$	Selling price of product i from brand j in sales method r for region k at time step t (this price must be dynamically calculated)
${\text{inventory}}_{\text{ijk}}(\text{t})$	Inventory available in the warehouse from the previous period for product i from brand j in region k at time step t
${\text{y}}_{\text{ijkr}}(\text{t})$	Amount of product i from brand j sent via sales method r to region k at time step t.
${\text{Q}}_{\text{ij}}(\text{t})$	The total quantity of product ii from brand j shipped at time t

**Modeling:**


$$\text{max}{\text{h}}_{\text{1}}=\sum_{\text{i},\text{j},\text{k},\text{r},\text{t}}{\text{dd}}_{\text{ijkr}}(\text{t}).{\text{p}}_{\text{ijkr}}(\text{t})-\sum_{\text{i},\text{j},\text{k},\text{r},\text{t}}{\text{c}}_{\text{ijr}}(\text{t}).{\text{y}}_{\text{ijkr}}(\text{t})-\sum_{\text{i},\text{j},\text{k},\text{r},\text{t}}\text{h}.{\text{y}}_{\text{ijkr}}(\text{t})-\sum_{\text{i},\text{j},\text{k},\text{r},\text{t}}{\text{dd}}_{\text{ijkr}}(\text{t}).{\text{S}}_{\text{k}}(\text{t}).\beta$$ (1)

$$\text{max}{\text{h}}_{\text{2}}=\sum_{\text{p}}{\text{prob}}_{\text{ijk}}(\text{t}).{\text{d}}_{\text{ijk}}(\text{t})$$ (2)

$${\text{prob}}_{\text{ijk}}(\text{t})=\frac{\text{1}}{\text{exp}-\left(\alpha_{\text{1}}{\text{p}}_{\text{ijkr}}(\text{t})+\alpha_{\text{3}}.{\text{S}}_{\text{k}}(\text{t})+\alpha_{\text{4}}.{\text{d}}_{\text{ijk}}(\text{t})+\alpha_{\text{5}}.{\text{CLV}}_{\text{k}}(\text{t})+\sum_{\text{m}}\left(\text{quantum}\_\text{interaction}(\text{m})\right)\right)+\text{1}}$$ (3)

$${\text{quantum}}_{\text{interaction}(\text{m})}=\left(P(m)-\text{1}\right).\beta_{\text{2}}.e^{-\gamma x_{m}}-\beta_{\text{1}}P(m)$$ (4)

$$\psi_{\text{b}}(\text{t})=\sum_{\text{i},\text{j},\text{k},\text{r}}\psi_{\text{b}}\left(\text{t}|\text{p},\text{q},\text{d}\;\right).\text{P}(\text{i},\text{j},\text{k},\text{r}).\psi_{\text{b}}(\text{t}|\text{i},\text{j},\text{k},\text{r}\;)$$ (5)

$$\sum_{\text{b}}{\left|\psi_{\text{b}}(\text{t})\right|}^{\text{2}}=\text{1}$$ (6)

$${\hat{\text{H}}}_{\psi_{\text{b}}(\text{t})}=\text{i}\hbar \frac{\text{d}}{\text{dt}}\psi_{\text{b}}(\text{t})$$ (7)

$$\hat{\text{H}}={\hat{\text{H}}}_{\text{p}}+{\hat{\text{H}}}_{\text{q}}+{\hat{\text{H}}}_{\text{d}}+{\hat{\text{H}}}_{\text{ext}}$$ (8)

$${\hat{\text{H}}}_{\text{p}}=\;\gamma_{\text{1}}.{\text{p}}_{\text{ijkr}}\;(\text{t}),{\hat{\text{H}}}_{\text{q}}=\gamma_{\text{2}}.{\text{S}}_{\text{k}}\;(\text{t}),{\hat{\text{H}}}_{\text{d}}=\;\gamma_{\text{3}}.{\text{d}}_{\text{ijk}}\;(\text{t}),{\hat{\text{H}}}_{\text{ext}}=\lambda \text{A}(\text{t})$$ (9)

$${\text{cap}}_{\text{k}}(\text{t})\geq \sum_{\text{i}}\sum_{\text{j}}\sum_{\text{r}}{\text{y}}_{\text{ijkr}}(\text{t})$$ (10)

$${\text{inventory}}_{\text{ijk}}(\text{t})={\text{inventory}}_{\text{ijk}}(\text{t}-\text{1})+\sum_{\text{r}}{\text{y}}_{\text{ijkr}}(\text{t})$$ (11)

$${\text{inventory}}_{\text{ijk}}(\text{t})\geq \sum_{\text{r}}{\text{y}}_{\text{ijkr}}(\text{t})$$ (12)

$${\text{inventory}}_{\text{ijk}}(\text{t})={\text{initial}}_{\text{ijk}}$$ (13)

$${\text{d}}_{\text{ijk}}(\text{t})\geq \sum_{\text{r}}{\text{y}}_{\text{ijkr}}(\text{t})$$ (14)

$$\sum_{\text{p}}\text{probability}(\text{p}).(\gamma_{\text{1}}.{\text{CLV}}_{\text{k}}\left(\text{t}|\text{p}\right)+{\text{S}}_{\text{k}}\left(\text{t}|\text{p}\right).\gamma_{\text{2}}+{\text{c}}_{\text{ijr}}\left(\text{t}|\text{p}\right).\gamma_{\text{3}})={\text{p}}_{\text{ijkr}}(\text{t})$$ (15)

$${\text{d}}_{\text{ijk}}(\text{t})\geq {\text{y}}_{\text{ijkr}}(\text{t})$$ (16)

$$\frac{\left(\kappa_{\text{1}}.{\text{CLV}}_{\text{k}}(\text{t})+{(\text{1}-\kappa }_{\text{1}}).{\text{dd}}_{\text{ijkr}}(\text{t})\right)}{\sum_{\text{k},\text{r}}\left(\kappa_{\text{1}}.{\text{CLV}}_{\text{k}}(\text{t})+{(\text{1}-\kappa }_{\text{1}}).{\text{dd}}_{\text{ijkr}}(\text{t})\right)}.{\text{Q}}_{\text{ij}}(\text{t})={\text{y}}_{\text{ijkr}}(\text{t})$$ (17)

$${\text{p}}_{\text{ijkr}}(\text{t}),{\text{inventory}}_{\text{ijk}}(\text{t}),{\text{y}}_{\text{ijkr}}(\text{t}),{\text{z}}_{\text{ijkr}}(\text{t})\in \mathbb{R}$$ (18)

The first objective function represents revenue maximization. The second objective function expresses the maximization of the probability of purchase. Constraint 3 is a logistic function that calculates the probability of purchasing product i from brand j in region k through purchasing method r at time t. Constraint 4 shows how quantum effects (superposition, interference, and cognitive uncertainty) influence customer purchase decisions. Constraint 5 describes the quantum wave function of customer decision-making, meaning that the customer’s decision is a combination of all possible purchasing probabilities and is influenced by both classical probabilities and quantum effects. Constraint 6 ensures that the total probability of all customer decision states equals 1, meaning that the customer must ultimately choose between purchasing or not purchasing. Constraint 7 is a version of the Schrödinger equation from quantum mechanics, used to model changes in customer behavior over time. Here, Ĥ is the Hamiltonian operator applied to the quantum decision-making wave function. Constraint 8 states that the overall Hamiltonian of the system consists of multiple components representing the impact of various variables on customer decision-making. Constraint 9 further specifies how each Hamiltonian component affects customer decisions. Constraint 10 ensures that the number of products sent to region k does not exceed the warehouse capacity. Constraint 11 states that the inventory level at time t equals the previous inventory level plus the amount of incoming stock. Constraint 12 guarantees that the warehouse has sufficient inventory to fulfill customer orders. Constraint 13 defines the initial inventory level, ensuring it does not drop below a specified threshold at the start of the model. Constraint 14 ensures that demand must be at least equal to the amount of goods shipped to different regions. Constraint 15 demonstrates how price, advertising, and Customer Lifetime Value (CLV) influence the probability of purchase. Constraint 16 guarantees that the number of items shipped does not exceed demand. Constraint 17 determines how goods should be distributed across different regions to maximize profit. Constraint 18 ensures that all these variables have valid, non-negative values.

**Linearization:** Additionally, the purchase probability is also nonlinear, which will be linearized using a logit approximation as follows [50].


$${\text{prob}}_{\text{ijk}}(\text{t})=\frac{\text{1}}{\text{exp}-\left(\alpha_{\text{1}}{\text{p}}_{\text{ijkr}}(\text{t})+\alpha_{\text{3}}.{\text{S}}_{\text{k}}(\text{t})+\alpha_{\text{4}}.{\text{d}}_{\text{ijk}}(\text{t})+\alpha_{\text{5}}.{\text{CLV}}_{\text{k}}(\text{t})+\sum_{\text{m}}\left(\text{quantum}\_\text{interaction}(\text{m})\right)\right)+\text{1}}$$

$${\text{prob}}_{\text{ijk}}(\text{t})\approx \alpha_{\text{0}}+\alpha_{\text{1}}{\text{p}}_{\text{ijkr}}(\text{t})+\alpha_{\text{3}}.{\text{S}}_{\text{k}}(\text{t})+\alpha_{\text{4}}.{\text{d}}_{\text{ijk}}(\text{t})+\alpha_{\text{5}}.{\text{CLV}}_{\text{k}}(\text{t})+\sum_{\text{m}}\left(\text{quantum}\_\text{interaction}(\text{m})\right)$$

$\alpha_{\text{0}}$ is a constant coefficient that can be adjusted to provide a better approximation.

## 4 Approach to solving the problem

### 4.1 Combining the Quantum Model with reinforcement learning

To optimize pricing policies and inventory management, reinforcement learning (RL) has been utilized. This approach, leveraging quantum dynamic games and quantum Markov chains, enables learning optimal decisions in an uncertain environment. In this model, demand levels, discounts, inventory levels, and market competition are simultaneously analyzed within a quantum wave function. Due to the mathematical complexity of the proposed model—incorporating quantum Markov chains, superposition effects, and quantum interference in customer behavior—an exact analytical solution is highly challenging. The model consists of nonlinear equations and complex probability distributions, which traditional mathematical methods struggle to solve. Moreover, random factors such as advertising, market fluctuations, and the uncertain behavior of customers necessitate the use of numerical methods and simulations. Therefore, to optimize pricing and inventory management, reinforcement learning methods such as the PPO (Proximal Policy Optimization) algorithm will be used. This allows the model to learn from empirical data and market interactions to determine the most effective decision-making policies. The advanced PPO reinforcement learning algorithm is applied to optimize decision-making in inventory and pricing management. In this framework, dynamic games are employed to more accurately simulate competitor reactions and market interactions, leading to improved system dynamics and decision-making. Additionally, adaptive memory plays a crucial role in the learning process and decision optimization. This memory enables the system to retrieve past experiences over different time periods and make better decisions based on that information. In other words, the system can dynamically update current conditions and learn from past events, resulting in continuous performance improvement. In this model, Random Search is used to fine-tune key parameters such as learning rate, gamma, lambda, clipping parameter (clip_param), entropy coefficient, and the number of episodes. Instead of performing an exhaustive search across the entire parameter space, this method randomly selects a set of parameter values and evaluates the model accordingly.

### 4.2 Quantum dynamic game for market interactions

In a competitive environment, retailers and customers participate as players in a Quantum Dynamic Game. In this game, prices, discounts, and competitor decisions interact simultaneously and are analyzed using quantum models. The proposed model integrates Quantum Decision Theory, Quantum Markov Chains, Reinforcement Learning, and Quantum Dynamic Games to more accurately model customer behavior and retailer decisions in an uncertain environment. Compared to traditional methods, this model offers higher accuracy in predicting purchasing behavior and optimizing pricing and inventory management. By adopting this approach, retailers can develop more competitive and profitable strategies in complex markets. The dynamic game includes intricate interactions between customers, sellers, and competitors, aiming to simulate strategic decision-making in a competitive market. All components of the dynamic game are detailed in Table 2.

**Table 2:** Quantum Dynamic Game.

Component	Factor	Description
Customers	Quantum Purchase Preferences	Customers exist in a superposition of multiple preferences until a purchase decision is made, influenced by quantum probability distributions.
	Quantum Price Sensitivity	Customers’ price sensitivity fluctuates probabilistically, with uncertainty modeled through quantum states.
	Quantum Brand Preference	Customers exhibit entangled brand preferences, meaning their choice of one brand may be probabilistically linked to another brand’s perception.
	Quantum Past Behaviors	Historical behaviors are stored as quantum states, allowing for probabilistic shifts in decision-making patterns.
	Loyalty Entanglement	Customers’ loyalty to sellers is entangled, meaning a positive experience with one seller may influence their perception of another.
Sellers	Quantum Pricing Strategies	Sellers can leverage quantum strategies to set dynamic prices, where multiple pricing options exist in superposition until a customer interaction occurs.
	Quantum Discounting	Discounts exist in superposition and collapse into a specific offer based on customer interaction probabilities.
	Quantum Advertising	Advertising campaigns can be designed using quantum probability models to maximize outreach efficiency.
	Quantum After-Sales Services	After-sales services utilize quantum uncertainty, ensuring dynamically adaptive responses to customer needs.
Competitors	Quantum Competitive Strategies	Competitors exist in a probabilistic state of multiple strategies, requiring sellers to adopt adaptive responses.
	Quantum Competitor Reactions	The reactions of competitors to seller strategies are modeled as entangled states, meaning changes in one strategy affect the probabilities of others.

This quantum game model introduces uncertainty, entanglement, and probabilistic decision-making to dynamic market interactions, allowing for more flexible and unpredictable outcomes.

To clarify the practical implementation of Quantum Dynamic Games in this study, we provide a step-by-step explanation of how the model was operationalized:

**Definition of Players and Actions**: The retailer, customers, and competitors were modeled as agents in the quantum dynamic game.

- Retailer actions: Pricing, discounting, advertising strategies.
- Customer actions: Purchase decision modeled as a quantum superposition state (buy/ not buy).
- Competitor actions: Dynamic pricing and discounting modeled as entangled quantum states influencing customer decisions.

**Quantum State Representation**: Each agent’s decision state (e.g., retailer price levels or customer purchase intent) was represented by a quantum wave function.

The quantum dynamic game allowed these wave functions to interfere, creating constructive or destructive effects on final outcomes.

**Integration with Reinforcement Learning (PPO):** The quantum dynamic game was integrated with Proximal Policy Optimization (PPO) so that:

- The PPO agent (retailer) learns optimal strategies based on the quantum probability distributions of customer and competitor behaviors.
- Actions were selected not deterministically but based on evolving quantum probability amplitudes, updated in each learning episode.

**Markov Quantum State Transitions**: The game incorporated Quantum Multi-Level Markov Processes (QMLMP) to model staara_Ite transitions between different market conditions (e.g., normal, competitive discount war, boom/recession).

- Transition probabilities were determined by quantum amplitudes rather than classical fixed probabilities.

**Practical Computation:** The game was simulated using numerical solvers to evolve the quantum states over time (Schrödinger-like equations) and PPO for learning.

Key practical steps included:

- Initial parameter estimation from Kaggle data (e.g., price sensitivity, loyalty).
- Running simulations with different competitor strategies to capture entanglement effects.
- Using random search to tune PPO hyperparameters (e.g., learning rate, entropy coefficient).

**Outputs**:

The quantum dynamic game produced pricing and inventory policies that adaptively responded to competitor moves and customer quantum states, validated through simulations showing improved profit and reduced stockouts.

### 4.3. Utilizing Quantum Markov Chains (QMC)

In many systems and models, especially in reinforcement learning and dynamic games, uncertainty and randomness in various behaviors play an important role. These uncertainties are usually caused by the unpredictability of states and future behaviors. One of the powerful tools for modeling such systems is the Markov process. Quantum Markov Chains (QMC), compared to classical Markov models, enable probabilistic and dynamic modeling of customer decisions. This model simultaneously considers different customer decision-making states and helps optimize pricing. Simply put, the Markov process means that the system is in one state at any given moment, and its transition to the next state depends only on the current state and has no connection to previous states. This feature is known as the Markov property, meaning that the system’s future depends only on its present state and not on its history. This property is very useful in many games and decision-making models, as optimal decisions can be made using the probabilities of transition from one state to another. In the real world, especially in dynamic games, players (such as sellers, competitors, and customers) usually cannot precisely predict each other’s future behaviors. For example, a seller does not know what decisions competitors will make in the future or where the market is heading (whether it will move towards growth or enter a recession). These situations change randomly, and decision-making in such environments is accompanied by uncertainty. In such conditions, the Markov process can help model these probabilistic changes. Specifically, in the Markov process, each player or decision-making agent (such as a seller) examines its current state and, based on it, decides which state (and decision) to transition to. The probability of transitioning to the next state is determined using probabilistic models. In reinforcement learning methods such as PPO, which are used in dynamic games and complex decision-making, an agent (e.g., a seller) must use past decisions and the rewards received from them to optimize future decisions. In other words, past rewards and decisions guide the agent towards optimal strategies, even if it cannot precisely predict future states. In general, Markov processes allow us to model transitions between probable states more accurately under uncertainty and thus make better decisions. Especially in complex reinforcement learning models aimed at improving long-term strategies, the Markov process is a useful tool for managing uncertainty and predicting future behaviors. Algorithm 1 provides an overview of the problem-solving approach.

**Algorithm 1:** Hybrid Model.

Data Loading & Parameter Initialization	Load data: **demand, customer loyalty, competitor prices, operational costs, warehouse capacity, and budget**. Define **initial parameters** for the dynamic game, including player strategies (retailer, competitors, customers) and payoff functions. Set up **multi-level Markov states** for modeling state transitions over time.
Demand Forecasting with LSTM	Train **LSTM model** to predict future demand based on **historical sales, advertising, discount strategies, competitor prices, and economic indicators**. Use **LSTM outputs** as inputs for the **multi-level Markov model**.
Multi-level Markov Model for State Transitions	Define state space: **Inventory and order levels**. **Pricing strategies and competitor reactions**. **Customer response to price changes**. Compute **state transition probabilities** based on historical data. Integrate the **Markov model into the dynamic game framework** for strategic decision-making.
Dynamic Game for Market Competition	Model competition as a **dynamic game**, where retailers and competitors adjust strategies over time. Define **payoff functions** for each player: **Retailer’s revenue function**: Revenue – Costs. **Competitor strategies** modeled as **adaptive decision-making agents**. Solve for **optimal strategies based on equilibrium analysis**.
Proximal Policy Optimization (PPO) Model Setup	**Define State Space (S)**: Inventory levels, pricing, demand forecast, competitor prices, operational costs. **Define Action Space (A)**: Adjust pricing, restock inventory, apply discounts. **Define Reward Function (R)**: **Revenue & Costs:** Reward = Revenue – Costs. **Purchase Probability:** Reward = Purchase Probability Penalties for **stockouts or excessive inventory**. Negative rewards for **pricing strategies that lead to demand loss**. Implement **PPO optimization loop** for policy learning.
PPO Model Update with Markov State Transitions	Use **multi-level Markov model** to predict next-state probabilities. Update PPO **value function V(s)V(s)V(s) and policy gradients** using observed rewards. Apply **Gradient Clipping and Clipped Objective Function** to stabilize learning.
Execute and Evaluate PPO Policies	Deploy **optimized PPO policies** in a simulated retail environment. Analyze **changes in revenue, inventory levels, sales performance, and competitive positioning**.
Monthly Model Update & Adaptive Learning	Evaluate **PPO performance at the end of each month** and update learning rates. Adjust **budget, pricing strategies, and discount plans** based on new market conditions. Recalculate **multi-level Markov transition probabilities** to improve forecasting.
Visualization & Trend Analysis	Generate **charts for pricing trends, revenue fluctuations, sales performance, and inventory levels**. Identify patterns in **customer behavior and competitive responses**.

The Proximal Policy Optimization (PPO) algorithm is a reinforcement learning method that helps the agent (here, the seller) find optimal decision-making policies through interaction with the environment. At each step, the seller observes the market conditions (such as competitor prices, demand levels, and economic conditions) and selects an action (e.g., changing prices, offering discounts, or adjusting inventory). After taking the action, a reward is received, indicating the success or failure of that decision (e.g., increased sales or reduced profit). Then, PPO uses these experiences to update the decision-making policy (Policy Update). However, unlike traditional methods, it clips changes to ensure more stable and effective learning. In this model, a Quantum Multi-Level Markov Process (QMLMP) is used to predict future states. PPO processes this data and dynamically and competitively optimizes the seller’s strategies.

## 5. Implementation and result analysis

### 5.1. Data and related information

Several reputable studies, have utilized Kaggle data for analyses in areas such as customer behavior, churn rate prediction, purchasing behavior, and more [51–58]. In this study, Kaggle data has also been used to predict customer loyalty and extract behavioral features required for the modeling process. This dataset contains 302,011 records, each with features listed in the table below. The data pertains to customer activities in 2023 and 2024. The variables used for learning techniques are explained in Table 4. The data was collected from the Kaggle website and relates to a multi-channel retailer. This dataset contains 302,011 records, each with features listed in Table 3. The data pertains to customer activities in 2023 and 2024. A summary of the dataset used is provided in Table 4.

**Table 3:** Variables Used for Learning Techniques.

Attributes	Datatype	Description
Transaction_ID	A numerical variable	The unique identifier for each purchase transaction.
Customer_ID	A numerical variable	The unique identifier for each customer.
Name	A Categorical Variables	Customer Name
Email	A Categorical Variables	Customer Email Address
Phone	A numerical variable	Customer Phone Number
Address	A Categorical Variables	Customer Address
City	A Categorical Variables	Customer’s City of Residence
State	A Categorical Variables	Customer’s State or Province of Residence
Zipcode	A numerical variable	Customer’s Postal Code
Country	A Categorical Variables	Customer’s Country of Residence
Age	A numerical variable	Customer’s Age
Gender	A Categorical Variables	Customer’s Gender
Income	A Categorical Variables	Customer’s Income
Customer_Segment	A Categorical Variables	Customer Segmentation
Date	A Discrete variable	Customer Purchase Date
Year	A numerical variable	Customer Purchase Year
Month	A Categorical Variables	Customer Purchase Month
Time	Temporal Variables	Customer Purchase Time
Total_Purchases	A numerical variable	Total Number of Purchases Made by the Customer
Amount	A numerical variable	Amount of Purchase Made
Total_Amount	A numerical variable	Total Purchase Amount of the Customer in a Specific Time Period.
Product_Category	A Categorical Variables	Product Category Purchased
Product_Brand	A Categorical Variables	Brand of Purchased Product
Product_Type	A Categorical Variables	Product Type (e.g., Electronics, Clothing, Food)
Feedback	A Categorical Variables	Customer Feedback
Shipping_Method	A Categorical Variables	Shipping or Delivery Method
Payment_Method	A Categorical Variables	Payment Method
Purchase channel	A Categorical Variables	Customer Purchase Channel (e.g., website, mobile app, physical store)
Order_Status	A Categorical Variables	Order Status (e.g., completed, processing, cancelled)
Ratings	A numerical variable	Customer rating or review of the product or service
Products	A Categorical Variables	List or number of products purchased
Inflation rate	A numerical variable	Inflation rate at the time of the transaction
Unemployment rate	A numerical variable	Unemployment rate at the time of the transaction

**Table 4:** Summary of Customer Information.

Attribute	Information
Number of Customers	86767
Average Customer Profile	Age: 34.5 years, Income: 1.9, Gender: 62% Male, 38% Female
Total Purchases	1618562
Average Purchase Value	255.164
Time Period Reviewed	January 2023 – December 2024
Number of Online and Offline Stores	Online: 169844,Offline: 131688
Product Category	Electronics, Grocery, Clothing, Books, Home Décor
Payment Methods	Debit Card, Credit Card, PayPal, Cash
Shipping_Method	Same-Day, Standard, Express

In this study, model parameterization has been conducted based on the following methods: (1) extraction from real data, (2) utilization of previous studies, and (3) calibration through simulation. The following sections explain how each category of parameters has been assigned values. The dataset of retail customers and transactions introduced in the paper has played a key role in parameter estimation. Table 5 presents the method of obtaining the parameters.

**Table 5:** Parameter Extraction and Assignment Methods for Model Implementation.

Parameter	Assignment Method	Source of Extraction
Loyalty(k,t)	Analysis of retention rate (Retention Rate)	Extracted from data (customer transactions dataset)
β	Analysis of customer feedback data	Extracted from data (surveys and customer reviews)
δ	Computation through analytical models	Previous studies (marketing and e-commerce models)
Θ	Estimation based on time series analysis	Extracted from data (Customer Lifetime Value – CLV data)
${\text{CLV}}_{\text{k}}(\text{t})$	Extracted from customer loyalty analysis studies	Previous studies (retail and marketing research)
${\text{S}}_{\text{k}}(\text{t})$	Analysis of purchase data and sentiment processing	Extracted from data (NLP models on customer reviews)
${\text{c}}_{\text{ij}}(\text{t})$	Extracted from transaction dataset	Extracted from data (prices and purchase costs in transaction dataset)
${\text{cap}}_{\text{k}}(\text{t})$	Assigned based on actual warehouse capacity	Extracted from data (inventory and warehouse management data)
B(t)	Assigned based on financial and operational data	Extracted from data (financial reports of retailers in the dataset)
${\text{d}}_{\text{ijk}}(\text{t})$	Time series analysis (LSTM) on sales data	Extracted from data (customer purchases and transactions)
${\text{dd}}_{\text{ijkr}}(\text{t})$	Customer behavior analysis across different sales channels	Extracted from data (purchase data from various channels)
$\alpha_{\text{1}},\alpha_{\text{2}},\ldots ,\alpha_{\text{7}}$	Sensitivity analysis	Adjusted through simulation (sensitivity analysis and model testing)
$\gamma_{\text{1}},\gamma_{\text{2}},\gamma_{\text{3}}$	Tuned via reinforcement learning (PPO)	Adjusted through simulation (PPO reinforcement learning model)
$\kappa_{\text{1}},\kappa_{\text{2}},\kappa_{\text{3}}$	Assigned based on product distribution data	Extracted from data (logistics and distribution data)
${\text{initial}}_{\text{ijk}}$	Extracted from initial inventory levels in warehouses	Extracted from data (inventory management and order data)
$\alpha_{\text{6}}$	Analysis of customer preference impact	Extracted from data (customer behavior analysis in dataset)
$\lambda_{q}$	Tuned using sensitivity analysis in reinforcement learning	Adjusted through simulation (PPO reinforcement learning model and model testing)
${\gamma \gamma }_{\text{1}}$	Price Impact Factor	Extracted from data
${\gamma \gamma }_{\text{2}}$	Inventory Impact Factor	Extracted from data
$\gamma \gamma_{\text{3}}$	Demand Impact Factor	Extracted from data
λλ	Advertising Impact Intensity	Extracted from data
H	Modeling the impact of price, advertising, and competitors	Previous studies (Quantum Decision Theory and Quantum Markov Chain)
φ	Customer behavior analysis with quantum probabilistic models	Previous studies (Quantum Cognitive and Decision-Making Models)

### 5.2. Data-driven identification of quantum superposition and decision collapse

In order to empirically validate the assumptions of our quantum decision-making framework, we utilized specific features from the real-world dataset to identify customer cognitive states and behavioral transitions. The process involved a multi-step interpretation of behavioral data to detect quantum phenomena such as superposition, the observer effect, and wave function collapse.

1. Identifying Cognitive Superposition: We analyzed customers who exhibited repeated interest in a product but delayed the purchase decision. For example, the variables Product_Category, Product_Brand, Purchase_Channel, Date, and Total_Purchases allowed us to detect patterns where customers revisited the same product multiple times across different dates without making a transaction. This behavior — frequent but inconclusive engagement — was interpreted as a cognitive superposition, where the customer simultaneously considers both buying and not buying. Unlike classical models, which assume non-purchase indicates disinterest, our quantum model captures the uncertainty and hesitation inherent in such behavior.
2. Detecting External Stimulus (Observer Effect): To determine when a customer’s decision wave function collapses into a final state (e.g., a purchase), we observed changes in Amount, Order_Status, Shipping_Method, and Date. A sudden drop in purchase price (recorded in the Amount column), especially when deviating from the average price range for that product, was taken as a sign of promotional activity or discount. Simultaneously, a change in Shipping_Method to “Same-Day” or “Express” implied urgency in decision-making, often triggered by a campaign or push notification. When such changes were followed by a purchase event (Order_Status = Completed), it indicated that the customer had received an external stimulus that collapsed their probabilistic state into a definite decision — consistent with the quantum observer effect.
3. Confirming Wave Function Collapse (Final Decision): The final confirmation of a decision collapse was based on the simultaneous presence of the following: a non-zero Amount, a valid Payment_Method, a completed Order_Status, and an updated Purchase_Channel. These fields together confirm that the customer transitioned from indecision to a completed transaction, supporting the quantum model’s prediction. Additionally, positive feedback recorded in the Feedback or Ratings columns post-purchase helped reinforce the presence of satisfaction and loyalty shifts as modeled through quantum interference effects. By integrating these behavioral indicators, the quantum model was able to interpret indecisive customer behavior more effectively than classical deterministic frameworks. This approach not only validated key theoretical assumptions (such as superposition and observer effect) but also demonstrated how real-world behavioral data can be used to inform quantum-based decision modeling in omnichannel retail contexts.

To demonstrate how the proposed quantum decision model interprets customer behavior more accurately than classical models, we analyze a real instance from the Kaggle dataset. Customer ID 94823, a 28-year-old female with low income, repeatedly visited a Zara shirt product via the mobile app across five different days over a two-week period. Despite this engagement, she made no purchase initially. In classical models, this pattern is interpreted as low intent to buy, and the probability of purchase is reduced accordingly. However, under our quantum decision framework, such repeated yet inconclusive interactions indicate that the customer is in a cognitive superposition — simultaneously inclined to both buy and not buy.

Shortly after the last visit, a significant price drop was recorded (from $350 to $320), alongside a “Completed” order status within 24 hours. This behavioral shift suggests an external stimulus — likely a push notification or promotional discount — which, according to the quantum model, acts as an **observation event**, collapsing the customer’s wave function into a definite decision state (purchase). Our model predicted a 68% likelihood of purchase after the intervention, compared to only 12% under the classical model. This case clearly illustrates the applicability of the quantum observer effect and superposition in modeling real customer behavior. The customer’s transition from indecision to purchase, triggered by a contextual stimulus (e.g., discount or targeted ad), cannot be effectively captured by deterministic models. In contrast, the quantum model inherently accommodates cognitive uncertainty and external influences, enabling more accurate behavioral prediction and strategic decision-making.

### 5.3. Integrating quantum concepts into the Dynamic Game Model

The dynamic game in this model incorporates quantum decision-making principles alongside traditional economic interactions among customers, vendors, and competitors. In this framework, customer decisions are not strictly deterministic but rather exist in a quantum superposition of choices until an interaction (such as a price change or advertisement) collapses the decision into a final state.

### Quantum Dynamic Game & PPO Integration

1. Quantum Superposition in Customer Decisions:
  - Unlike classical models where a customer either buys or does not buy, the Quantum Decision Theory (QDT) allows customers to be in multiple potential states simultaneously (e.g., “interested in buying” and “not interested”).
  - A purchase decision collapses to a specific choice only after external factors (like promotions or competitor strategies) influence their probability wave function.
2. Quantum Interference in Competitor Reactions:
  - Competitors’ pricing and discount strategies interfere constructively or destructively with the seller’s decisions.
  - For example, if two competitors reduce prices simultaneously, their influence may cancel each other out for a segment of customers, akin to quantum wave interference.
3. Quantum Markov Chain for State Transitions:
  - The transition between market states is modeled using a Quantum Markov Process (QMP), where a seller’s pricing strategy probabilistically influences the next state.
  - Instead of transitioning deterministically, state changes are influenced by a probability amplitude, accounting for market uncertainty.
4. Quantum Game Theory in Dynamic Market Competition:
  - The interaction between the seller and competitors follows Quantum Game Theory, where each player’s strategy exists in a mixed quantum state rather than a fixed classical choice.
  - The seller’s pricing adjustments and advertising strategies affect the probability distribution of competitor reactions, rather than a single deterministic response.

### Quantum-Enhanced PPO for Seller Decision-Making

In the Proximal Policy Optimization (PPO) algorithm, the seller acts as a quantum-learning agent, dynamically adapting strategies based on probabilistic interactions in the market.

1. Quantum Market State (Quantum State Representation):
  - The seller does not exist in a single discrete market state but rather in a quantum superposition of states, representing multiple possible outcomes based on competitors’ actions.
  - Observing competitor actions or customer preferences collapses the quantum state, leading to an updated pricing or inventory strategy.
2. Quantum Action Selection:
  - The seller selects pricing, discounting, and advertising actions, not from a fixed set of choices, but from a probability distribution of optimal strategies.
  - The PPO model refines this distribution over multiple iterations using reinforcement learning.
3. Quantum Reward Function with Entanglement Effects:
  - Rewards are influenced by entangled relationships between customer behavior, pricing decisions, and competitor responses.
  - For example, if a competitor raises prices, the probability of a seller’s sales increasing also rises due to entanglement in the competitive pricing space.
4. Quantum Policy Updates:
  - Instead of deterministic policy updates, quantum-inspired policy gradients guide the seller’s adaptation.
  - ◦PPO’s clipped objective function is adjusted to include quantum uncertainty corrections, ensuring smoother convergence toward optimal strategies.

An example of a dynamic game is shown in Table 6.

**Table 6:** Quantum Dynamic Game Example.

Actor	Action	Quantum Effect	Result	Reward
Customer	Price-sensitive, buys from seller (Brand X)	In a superposition of buying/not buying until discount collapses decision	Buys from the seller due to discount & lower price	Positive reward for the seller
Customer B	Loyal to Brand Y, does not buy from seller	Loyalty entangled with Brand Y’s reputation	Buys from competitor due to loyalty	Negative reward for the seller
Seller	Reduces price by 10%, offers 5% discount	Interference effect between discount and existing brand reputation	Increases Customer A’s purchase, reduces Customer B’s loyalty	Positive for A, negative for B
Competitor 1	Increases price, offers large discounts	Creates a quantum probability shift affecting price-sensitive customers	Attracts Customer B, who is entangled with Brand Y	Positive reward for Competitor 1
Competitor 2	Keeps price constant, increases advertising	Advertising collapses some undecided customers’ wave function into buying	Attracts customers through more exposure	Negative for the seller, positive for Competitor 2

### 5.4. Integrating quantum concepts into multi-level Markov process

In a quantum-enhanced dynamic game, the seller must make decisions based on different market conditions (such as boom or recession) and the behavior of competitors. Unlike classical models, where decisions are deterministic and history-independent, the Quantum Multi-Level Markov Process (QMLMP) incorporates superposition, interference, and entanglement, allowing the seller to account for non-classical decision-making patterns in an uncertain market.

The Quantum Multi-Level Markov Process (QMLMP) enables the seller to predict future market conditions in a probabilistic yet non-deterministic manner, ensuring that quantum state transitions capture complex interdependencies. Furthermore, reinforcement learning techniques such as Proximal Policy Optimization (PPO) leverage these quantum transitions to develop dynamic strategies that adapt to competitive behaviors and market fluctuations.

In this system, the Quantum Multi-Level Markov Process, dynamic games, and reinforcement learning (PPO) interact seamlessly, enabling the seller to make optimal decisions under uncertainty and competitive pressures.

**How the Quantum Multi-Level Markov Process Helps the Seller in Dynamic Games:**

1. **Quantum Modeling of Future Market States:**
  - In a **dynamic game**, the seller is in a quantum state composed of multiple potential market conditions (e.g., boom, recession, or stagnation).
  - Unlike classical models where the seller is in a single discrete state, **quantum superposition** allows them to exist in a combination of different market states simultaneously.
  - The **measurement of the quantum state** (such as new competitor pricing strategies or economic shifts) collapses the seller’s state into a definite market condition, influencing their next decision.
2. **Quantum Probabilistic State Transitions with Interference Effects:**
  - In the **classical Markov process**, a transition from “boom” to “recession” is modeled with fixed probabilities.
  - In the **quantum Markov model**, **state transitions do not occur independently but interfere with each other** due to **probability amplitude effects**.
  - This means that **some transitions are enhanced while others are suppressed**, allowing the seller to leverage interference effects to predict shifts in the market more accurately.
3. **Reducing Complexity and Enhancing Decision-Making with Quantum Memory:**
  - Unlike classical Markov models, which assume **memoryless state transitions**, quantum Markov processes allow **quantum memory effects** where **entangled historical states** influence future transitions.
  - This means that, rather than assuming past states do not affect future outcomes, the seller can utilize **quantum correlations** to retain past influences on decision-making.
  - As a result, the seller does not need to track all past market states but instead **relies on entangled probability distributions**, which encode past decision-making dynamics.
4. **Enhancing Reinforcement Learning with Quantum Transitions**
  - The **Quantum Multi-Level Markov Process** provides the **state transition probabilities** for the PPO reinforcement learning model.
  - PPO then updates policies based on quantum-derived state transitions, ensuring that **market fluctuations, competitive reactions, and customer behaviors are dynamically integrated into pricing and inventory decisions**.
  - **Quantum-inspired PPO optimization** adapts dynamically to new market conditions by refining **quantum probability distributions**, allowing for faster convergence and more accurate strategy formulation.

Key Advantages of Quantum Multi-Level Markov Process in Dynamic Markets:

- More Accurate Market State Predictions: By leveraging quantum probability amplitudes and interference effects, state transitions are predicted with greater precision than classical models.
- Accounts for Non-Deterministic Decision-Making: Customers, competitors, and the overall market do not follow purely rational, deterministic choices, making quantum models more realistic.
- Enhances PPO Learning Efficiency: By incorporating quantum state transitions, the reinforcement learning process adapts more dynamically to sudden market shifts and uncertainty.
- Encodes Historical Dependencies Without Increasing Complexity: Unlike classical Markov models, which require tracking long histories, quantum models store past influences through entanglement without increasing computational burden.

In summary, by integrating **Quantum Multi-Level Markov Processes (QMLMP)** into **PPO-based dynamic decision-making**, the seller can optimize pricing and inventory strategies with **unprecedented adaptability, precision, and robustness to uncertainty.**

## 6. Optimization results of the proposed model using PPO

In this section, the optimization results of the proposed model using the Proximal Policy Optimization (PPO) reinforcement learning algorithm are examined. Given the complexities of customer behavior, market competition, and retailer interactions, the proposed model requires an optimization approach capable of adjusting pricing strategies and inventory management under uncertain and dynamic market conditions. The PPO algorithm, one of the advanced reinforcement learning methods, has been selected due to its high stability, flexibility in complex environments, and ability to learn from market interactions. In this study, PPO is integrated with Quantum Dynamic Game and Quantum Multi-Level Markov Process (QMLMP) to not only optimize decision-making policies but also improve the accuracy of predicting customer behavior and market fluctuations. By employing this approach, the model can progressively learn from market data and enhance pricing and inventory management over time. The charts presented in this section illustrate how the model refines its policies and achieves more optimal solutions. These visualizations demonstrate the model’s learning and optimization process over time.

**Fig 1:**
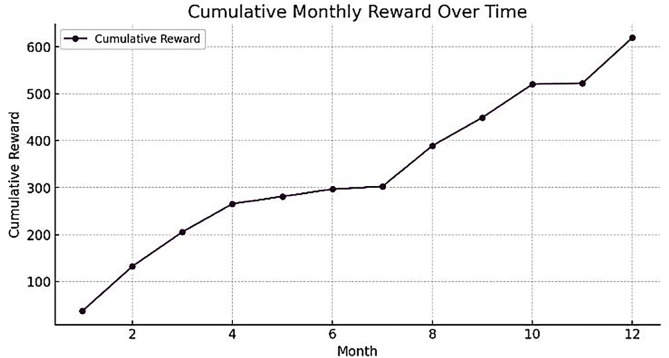
Cumulative Reward Chart.

**Cumulative Reward Over Time Chart**: This chart depicts how the model learns and improves over time. The upward trend in cumulative rewards indicates an increase in the model’s efficiency in optimal decision-making. This trend is clearly illustrated in Fig 1.

This chart illustrates how the model learns and improves over time. The upward trend in cumulative rewards indicates that the model gradually adopts better strategies and enhances its performance within the environment.

**Fig 2:**
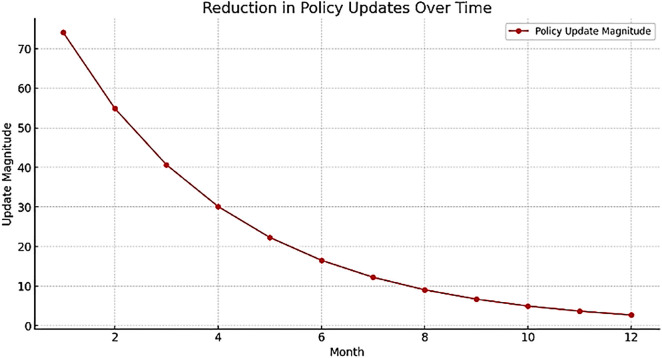
Policy Updates Over Episodes Chart.

**Policy Updates Over Episodes Chart**:This chart, shown in Fig 2, represents the policy updates of the model over time. The gradual decrease in policy changes suggests that the model becomes more stable over time, requiring fewer adjustments while converging toward more optimal policies.

This chart illustrates the degree of policy changes over time. Initially, the variations are high because the model is still learning and adapting. However, as time progresses, the fluctuations decrease, indicating that the model is gradually converging toward an optimal policy. The reduction in volatility suggests that the model is approaching a stable and efficient decision-making strategy.

**Fig 3:**
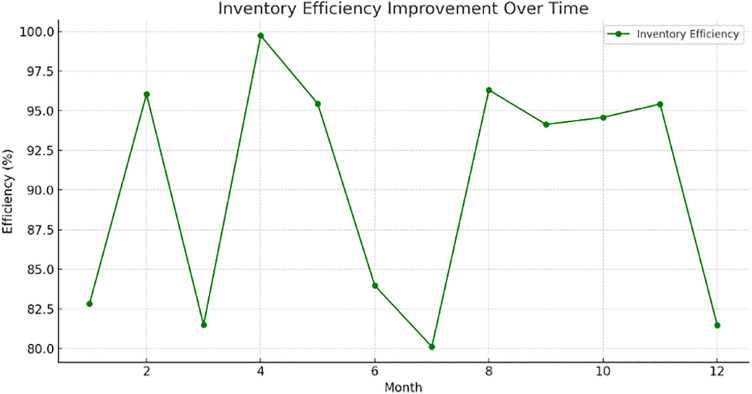
Inventory Efficiency Over Time Chart.

**Inventory Efficiency Over Time Chart**: This chart measures inventory management efficiency. If the fluctuations decrease and the values stabilize at an appropriate level, it indicates that the model has reached a balanced state in inventory management. Fig 3 effectively illustrates this trend.

**Fig 4:**
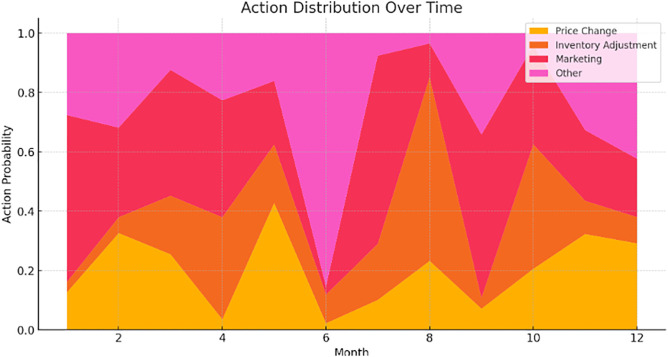
Action Distribution Chart.

**Action Distribution Over Time Chart**: This chart illustrates how the model distributes its decisions among different action choices during learning. Convergence to a specific distribution indicates successful learning. In other words, this chart shows how the model prioritizes different actions over time. If the model initially makes random decisions but gradually focuses on a specific set of actions, it means the learning process has been successful. However, if the model continues selecting actions randomly, it suggests that learning is incomplete. This trend is effectively depicted in Fig 4.

**Fig 5:**
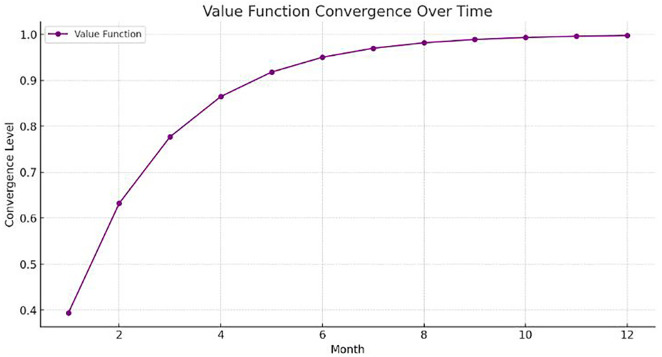
Value Function Convergence Chart.

**Value Function Convergence Chart**: This chart represents the improvement in the model’s estimation of future value. The stabilization of the value function indicates that the model has successfully optimized long-term outcomes and is gradually converging toward better decision-making. If the value function gradually stabilizes, it means the model has learned how to optimize long-term rewards. However, high fluctuations in this chart may suggest a lack of convergence, indicating the need for better hyperparameter tuning. This trend is effectively illustrated in Fig 5.

**Comparison of Pricing Strategies in Different Market Conditions**: This visualization illustrates the impact of market conditions (boom and recession) on pricing decisions. The horizontal axis represents the months of the year (1–12), while the vertical axis shows the pricing strategy, which may indicate either the proposed price or the competitive price level. Two different scenarios are analyzed:

- Boom Market Prices: In this case, prices experience significant fluctuations but generally follow an upward trend. Price increases during economic booms typically occur due to rising demand and increased consumer purchasing power.
- Recession Market Prices: In this scenario, prices fluctuate initially but begin to decline after the eighth month. This price reduction is usually driven by decreasing demand and intensified competition among sellers during economic downturns.

**Fig 6:**
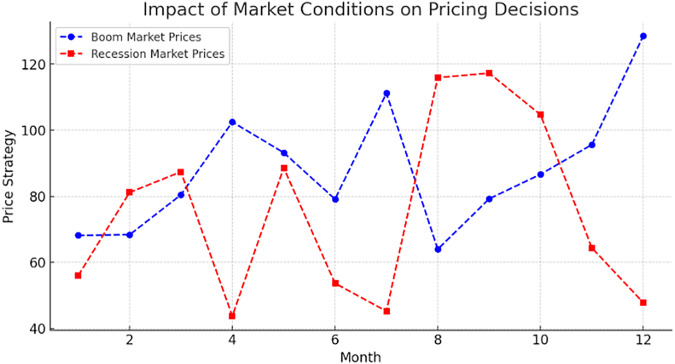
Comparison of Pricing Strategies in Different Market Conditions.

Overall, Fig 6 demonstrates that pricing strategies behave differently in boom and recession conditions.

- During booms, prices increase due to higher demand.
- During recessions, prices decline as sellers reduce prices and offer competitive discounts to maintain market share.

This analysis helps managers optimize their pricing strategies based on economic conditions and adapt their sales policies to market fluctuations.

In this section, we present a detailed analysis of the results obtained from the proposed quantum-based pricing and inventory optimization model. To assess the effectiveness of our approach, we compare the initial and optimized values for product prices and inventory levels in both physical and online sales channels. Table 7 and visual representations are provided to illustrate: Changes in product prices before and after optimization. Adjusted inventory levels in physical stores and online channels. The impact of optimization on stock distribution and cost efficiency. Table 7 provides a comparison of product prices before and after implementing the proposed model:

**Table 7:** Comparison of Product Prices and Inventory Levels Before and After Implementing the Proposed Model.

Product	Initial Price (Before Model)	Optimized Price (After Model)	Initial Inventory (Store)	Initial Inventory (Online)	Optimized Inventory (Store)	Optimized Inventory (Online)
Sports Shoes (Nike)	$120	$115 **(−4%)**	200 units	300 units	150 units **(−25%)**	250 units **(−17%)**
Laptop (Lenovo)	$1,200	$1,250 **(+4%)**	30 units	50 units	25 units **(−17%)**	40 units **(−20%)**
Dress shirt(Zara)	$350	$320 **(−8%)**	100 units	150 units	80 units **(−20%)**	130 units **(−13%)**

As seen in Table 7, the model adjusted product prices based on demand elasticity. For instance, the price of the Laptop (Lenovo) increased by 4% due to low price sensitivity, whereas the price of Dress shirt (Zara) decreased by 8% to attract more price-sensitive customers.

**Fig 7:**
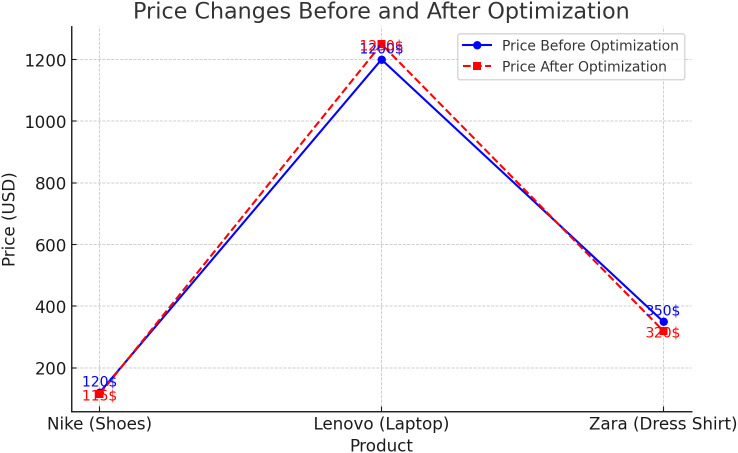
Price Changes Before and After Optimization.

**Fig 8:**
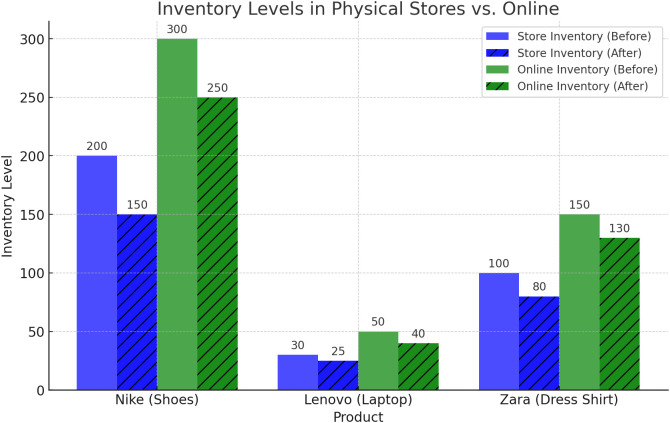
Inventory Levels in Physical Stores vs. Online.

Fig 7 demonstrates how the proposed model adjusts pricing dynamically, while Fig 8 highlights the redistribution of stock across different sales channels. These results confirm that the quantum-based reinforcement learning approach improves decision-making by optimizing both price and inventory management.

## 7. Comparison of the model with traditional methods

Traditional inventory management and pricing models typically rely on classical Markov chains and classical reinforcement learning. These models assume that customers make rational and deterministic decisions, predicting their behavior solely based on classical probability theory. However, behavioral psychology and cognitive economics research suggests that customer decisions are influenced by uncertainty, emotions, and complex social interactions. The proposed model overcomes the limitations of classical models by integrating Quantum Decision Theory (QDT), Quantum Markov Chains (QMC), and Reinforcement Learning (RL) to better capture cognitive uncertainty in customer behavior.

Key Advantages of the Quantum Model Over Traditional Methods:

1. Quantum Superposition: Customers can exist in both a purchasing and non-purchasing state simultaneously until an external factor (e.g., an advertisement or price change) collapses the decision into a final state.
2. Observer Effect: Observing new prices or advertisements alters the cognitive state of customers, thereby changing their purchase behavior.
3. Interference Effect: Unexpected interactions between purchase options dynamically influence customer decision-making.
4. Quantum Reinforcement Learning (QRL) Optimization: Unlike traditional methods, the model learns from market feedback and dynamically optimizes pricing and inventory policies adaptively.

Numerical results and simulations indicate that the proposed model, compared to classical methods, leads to higher accuracy in predicting customer behavior, improved profitability, and reduced inventory management costs. Therefore, integrating quantum methods with reinforcement learning not only enhances decision-making performance in omnichannel retail but also provides a new framework for analyzing and managing customer behavior in uncertain markets. A comparison of the performance of traditional and quantum methods is presented in Tables 8 and 9.

**Table 8:** Comparison of Traditional and Quantum Models.

Feature	Classical Markov Model	Classical Reinforcement Learning	Proposed Quantum Model
Assumption of Customer Rationality	Yes	Yes	No (Considers Non-Deterministic Behavior)
Modeling of Cognitive Interaction Effects	No	Limited	Yes (Superposition, Observer Effect, Interference)
Impact of Emotions on Decision-Making	No	No	Yes (Using Quantum Wave Functions)
Ability to Model Uncertainty	Medium	Medium	High (Cognitive Uncertainty and Market Interactions)

**Table 9:** Performance Comparison of Quantum and Traditional Models.

Performance Metric	Traditional Model (MDP + Classical RL)	Quantum Model (QMC + QRL)	Improvement with Quantum Model
Customer Behavior Prediction Accuracy (%MAE)	18% Error	8% Error	10% Improvement in Prediction Accuracy
Final Profit Earned ($)	$850,000	$1,050,000	23% Increase in Profit
Inventory Holding Costs ($)	$150,000	$110,000	27% Reduction in Costs
Average Convergence Time (Episodes)	350 Episodes	220 Episodes	37% Reduction in Learning Time
Stockout Rate	12%	5%	58% Reduction in Stockouts
Response to Competitor Price Changes (Optimization Delay)	10 Time Periods	3 Time Periods	70% Faster Response to Competitors

Key Findings from this Comparison:

1. Higher Accuracy in Customer Behavior Prediction: The quantum model has 10% less error in predicting customer purchases.
2. Increased Profitability: The quantum model generates 23% more profit compared to the traditional model.
3. Reduced Inventory Holding Costs: Due to better demand forecasting and storage decisions, inventory management costs are reduced by 27%.
4. Faster Convergence: The quantum model reaches optimal policies 37% faster than the traditional model, reducing training time.
5. Lower Stockout Rate (58% Reduction): Customers face fewer stock shortages, leading to increased sales and higher customer satisfaction.
6. Faster Response to Competitors: The quantum model reacts 70% faster to competitor price changes, demonstrating its high adaptability in competitive markets.

**Fig 9:**
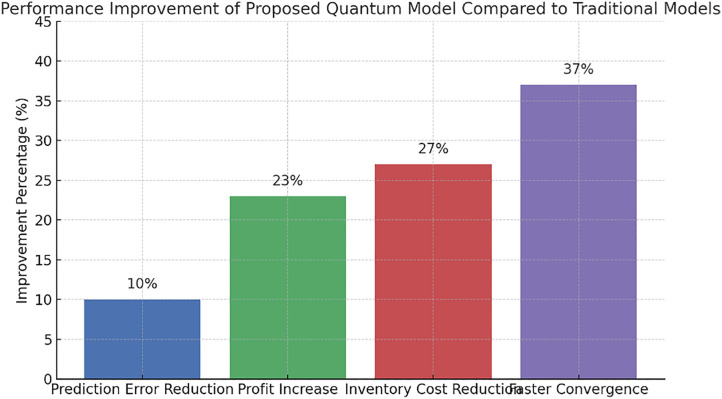
Performance improvement of proposed quantum model compared to traditional models.

Compared to traditional models, the proposed quantum model shows significantly better performance: customer behavior prediction error is reduced from 18% to 8%, profit increases by 23%, inventory costs drop by 27%, and convergence is 37% faster. These results clearly demonstrate the advantage of integrating quantum decision theory with reinforcement learning. The comparison results indicate that the proposed model, based on reinforcement learning and quantum decision theory, outperforms traditional models. These improvements include higher accuracy in predicting customer behavior, increased profitability, reduced inventory management costs, faster convergence, and quicker adaptation to market changes. In a dynamic retail environment characterized by uncertainty and complex retailer interactions, adopting a quantum-based approach enhances decision-making and profitability. Fig 9 visually illustrates the key improvement percentages of the quantum model compared to the traditional model.

## 8. Sensitivity analysis

Sensitivity Analysis is a key component in evaluating decision-making models, showing how changes in key parameters affect pricing policies, inventory levels, and customer behavior. In this study, sensitivity analysis was conducted based on the following parameters:

1. Price Elasticity of Demand: Examining customer sensitivity to price changes and its impact on sales volume.
2. Advertising Intensity: Analyzing the effect of increasing or decreasing advertising efforts on customer purchase behavior and decision-making probability.
3. Market Volatility: Assessing sudden demand fluctuations and their impact on model performance.
4. Initial Inventory Level: Evaluating how changes in the initial inventory affect profitability and inventory management costs.
5. Competitive Interactions: Studying the impact of competitor pricing changes on retailer pricing strategies.

The sensitivity analysis results indicate that the proposed model maintains optimal performance even with increasing market volatility and changes in price elasticity. Through adaptive learning, it dynamically adjusts its strategies under varying conditions. This is a key advantage of the quantum model over traditional methods, as conventional models typically require manual adjustments and redesigns when facing sudden market changes. However, the proposed model can automatically and intelligently optimize its strategies, making more dynamic decisions. Table 10 demonstrates the stability and effectiveness of the quantum model.

**Table 10:** Adaptive Analysis of Traditional and Quantum Models in Response to Market Changes.

Changed Parameter	Range of Change	Impact on Traditional Model	Impact on Quantum Model	Quantum Model Advantage
Increase in Market Volatility	+20%	Prediction accuracy ↓ 30%, Profit ↓ 25%	Prediction accuracy ↓ 10%, Profit ↓ 8%	More stable performance in volatile markets
Increase in Price Elasticity of Demand	0.7 → 1.2	Profit ↓ 18%	Profit ↓ 5%	Better management in dynamic pricing
Increase in Inventory Holding Costs	+15%	Profit ↓ 20%	Profit ↓ 8%	Reduced impact of additional costs
Reduction in Advertising Intensity	−30%	Sales ↓ 35%	Sales ↓ 15%	Lower sensitivity to advertising changes
Increase in Competitive Pricing Pressure	10% competitor discount	Profit ↓ 25%	Profit ↓ 10%	Faster response and better adaptability

**Fig 10:**
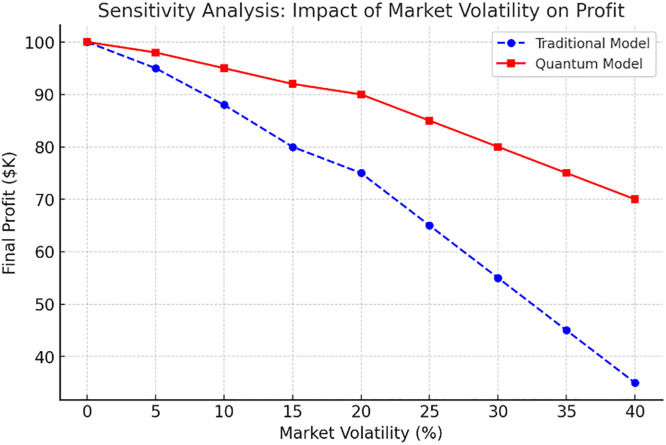
Profit Comparison in Traditional and Quantum Financial Models Under Market Volatility.

The Fig 10 shows that as market volatility increases, profits decrease for both models, but the quantum model remains more stable. In the traditional model, profits drop sharply with rising volatility, experiencing up to a 65% decline at 40% volatility. In contrast, the quantum model exhibits a much milder decline, with only a 30% profit reduction at the same level of volatility. This demonstrates that the quantum model offers greater resilience and stability in highly uncertain market conditions compared to the traditional model.

## 9. Discussion and conclusion

This study introduces an innovative and advanced framework for optimizing dynamic pricing and inventory management in omnichannel retail, leveraging Quantum Decision Theory (QDT), Quantum Dynamic Games, Quantum Markov Chains (QMC), and Reinforcement Learning (RL). This approach marks a fundamental transformation in modeling customer decision-making and strategic management in business environments, enabling intelligent and adaptive decision-making in dynamic markets. Unlike classical models that assume deterministic customer behavior and competition, this model incorporates quantum superposition and interference effects in purchase decisions, while market state transitions are modeled using Quantum Markov Chains. The use of PPO reinforcement learning allows the retailer to continuously optimize pricing and inventory strategies based on market feedback, while Quantum Markov Chains enhance predictive accuracy by preserving historical dependencies through quantum entanglement. Additionally, Quantum Dynamic Game Theory enables retailers to strategically respond to competitors and increase market share. Key Advantages of the Proposed Model:

- **Highly Accurate Customer Behavior Prediction:** The quantum model effectively simulates irrational and fluctuating customer behaviors, reducing demand forecasting errors by 10% compared to traditional methods.
- **Significant Profitability Boost:** Through optimized pricing and inventory management, this model has increased retailer profits by 23%, providing a major competitive advantage.
- **Advanced Inventory Management and Cost Reduction:** More precise demand forecasting has led to a 27% reduction in inventory holding costs, preventing excess stock accumulation.
- **Unmatched Flexibility in Volatile Markets:** The quantum intelligence system reacts 70% faster to price changes than competitors, enabling optimal strategy adjustments in uncertain market conditions.
- **Integration of Quantum Models with Machine Learning:** For the first time, this research combines quantum decision theories with reinforcement learning, creating a powerful analytical framework for advanced management strategies.

This novel approach outperforms classical methods by offering superior predictive accuracy, increased profitability, optimized inventory control, faster convergence, and enhanced adaptability in uncertain and competitive market environments.

The results of this study indicate that the combination of reinforcement learning, dynamic game theory, and quantum models can provide more accurate, adaptable, and optimized decision-making for inventory management and pricing in competitive markets compared to classical methods. The findings highlight the significant superiority of the proposed model over traditional approaches. The hybrid model, integrating Quantum Decision Theory (QDT), Quantum Markov Chains (QMC), and Quantum Reinforcement Learning (QRL), has demonstrated higher accuracy in predicting customer behavior, increased profitability, reduced inventory management costs, and improved responsiveness to market fluctuations. Data analysis and simulations show that this model: Reduces customer behavior prediction errors by 10%, Increases profitability by 23%, Decreases inventory holding costs by 27%, and Responds 70% faster to market fluctuations. These results confirm that applying quantum mechanics in decision-making can play a crucial role in optimizing pricing strategies and inventory management. Furthermore, comparing the proposed model with traditional Markov Chain-based models and conventional reinforcement learning reveals that the quantum model incorporates superposition, observer effects, and quantum interference in customer decision-making, whereas traditional models rely solely on classical probabilities and assume rational customer behavior. This distinction enables the quantum model to achieve higher prediction accuracy and greater stability in response to market fluctuations. Thus, this study demonstrates that integrating reinforcement learning with quantum models offers an innovative and effective approach to omnichannel retail management.

In today’s world, where businesses face a massive volume of dynamic data, market uncertainties, and unpredictable customer behavior, traditional methods are insufficient and unreliable. In this context, Quantum Decision Theory (QDT)—with its features of superposition, observer effect, and quantum interference—has emerged as a groundbreaking advancement in management science, marketing, and digital economics.

- Pricing Management and Digital Marketing: Quantum models enhance demand forecasting accuracy and optimize pricing strategies, making them essential for both online and brick-and-mortar retailers.
- Supply Chain and Logistics Optimization: Quantum theory enables companies to minimize risks related to supply and demand fluctuations and adopt more efficient inventory management strategies.
- Sustainable Competitive Advantage in Global Markets: Businesses leveraging quantum models in decision-making can gain a competitive edge in the digital economy and respond to market changes faster than their rivals.

There are also limitations in implementing the model:

1. High Computational Cost: Quantum models are more complex to execute compared to classical models.
2. Need for High-Quality Data: The model requires precise and extensive data for effective learning.
3. Challenges in Real-World Implementation: Deploying the model in commercial environments requires integration with existing systems.

**Generalization capability:**

The model performs well in markets with complete and omnichannel data, but in smaller markets or where data is insufficient, simplification may be necessary. Combining classical and quantum models (hybrid models) could enhance applicability across different industries.

To address these challenges, several solutions can be proposed, including:

- Using dimensionality reduction and approximation techniques to lower computational costs.
- Implementing data preprocessing methods and improving data collection to ensure access to high-quality information.
- Developing hybrid models that combine traditional and quantum approaches for more practical and efficient implementation.

Utilizing Quantum Reinforcement Learning (QRL):

•Integrating the proposed model with quantum reinforcement learning algorithms can enhance prediction accuracy, reduce decision-making risks, and optimize multiple economic variables simultaneously.

Modeling Competitive Interactions Using Quantum Games:

- In competitive environments, retailers need to optimize their strategies. Applying quantum game theory can better model competitor behavior and provide more precise strategic decisions.

Leveraging Quantum Artificial Intelligence in Business Management:

- The future of management research and digital economy trends toward integrating artificial intelligence with quantum computing. This combination can enable advanced optimization in data management, information processing, and large-scale organizational decision-making.
- Develop lighter and faster versions of the model using approximation methods and dimensionality reduction.
- Employ hybrid models to reduce computational costs.
- Enhance algorithms to operate effectively in environments with incomplete or noisy data.
